# Adrenocorticotropic Hormone-producing Extrapulmonary Small Cell Carcinoma of the Breast

**DOI:** 10.7759/cureus.6488

**Published:** 2019-12-28

**Authors:** Suha Abu Khalaf, Abdallah M Mansour, Harleen Chela

**Affiliations:** 1 Department of Internal Medicine, University of Missouri, Columbia, USA

**Keywords:** extrapulmonary small cell carcinoma, ectopic acth syndrome, breast cancer, hypokalemia, hypokalemic metabolic alkalosis

## Abstract

Extrapulmonary small cell carcinoma (EPSCC) of the breast is a very rare tumor. Adrenocorticotropic hormone (ACTH) production from these tumors is extremely rare and seldom reported resulting in significant diagnostic and therapeutic challenges and delays. We present a case of a 38-year-old female who presented with a breast lump and was diagnosed with primary EPSCC of the breast. Eighteen months later, she presented with refractory hypokalemia and metabolic alkalosis. Ectopic ACTH production was found to be the reason for her metabolic derangement. Extensive diagnostic evaluation, including a comprehensive metastatic workup, is necessary to differentiate this tumor from other primary malignancies such as lung and other breast cancers. We review the literature and discuss the diagnostic approach and suggested therapies.

## Introduction

Small cell carcinoma (SCC) is an aggressive malignancy that is thought to arise from multipotent stem cells that are capable of divergent differentiation. It was initially described as a primary lung malignancy. However, reports of SCC involving other organs with no pulmonary involvement were cited in the literature as early as 1930 [1]. These organs include urinary bladder, breast, skin, prostate, rectum, esophagus, colon, gallbladder, stomach, larynx, cervix, and salivary glands [2].

The reported incidence of extrapulmonary small cell carcinoma (EPSCC) in the United States ranges between 0.1% and 0.4% of all malignancies and up to 5% of SCC cases [2]. Primary breast EPSCC was first reported in 1963 [3]. Since then, few case reports and case series have reported this entity [4]. These tumors pose a diagnostic and therapeutic challenge due to the sparsity of published management protocols, poor pathological differentiation, histological mimicry to other primary malignancies, and their poorly understood aggressive nature and prognosis [2,5].

## Case presentation

A 38-year-old female with no relevant medical history presented to the oncology clinic with a right breast lump of one-month duration. She denied any associated pain, bleeding, discharge, itching, or skin changes. Physical exam was unremarkable except for a 2 cm hard lump palpable at the right upper quadrant of the right breast and a palpable axillary lymph node, which was mobile, hard, and about 1 cm x 1 cm in size. Basic blood work was unremarkable (Table 1).

**Table 1:** Initial Lab values upon diagnosis ANC: absolute neutrophil count; BUN: blood urea nitrogen; Cl^-^: chloride; HCO_3^-^_: bicarbonate; INR: international normalized ratio; K^+^: potassium; MCV: mean corpuscular volume; Na^+^: sodium; PTT: partial thromboplastin time; WBC: white blood cell.

Lab test (unit)	Result	Reference value
Complete blood count		
Hemoglobin (g/dl)	14.1	12-15.5
Hematocrit (%)	42	34.9-44.5
MCV (fl)	85	81.6-98.3
WBC (× 10^9^/l)	5.08	3.5-10.5
ANC (× 10^9^/l)	4.08	1.7-7
Platelets count (x10^9^/l)	199	150-450
Complete metabolic panel and miscellaneous		
Creatinine (mg/dl)	0.64	0.5-1.2
BUN (mg/dl)	7	6.0-20
Na^+^ (mmol/l)	139	136-145
K^+^ (mmol/l)	3.9	3.5-5.1
Cl^-^ (mmol/l)	101	92-107
HCO_3^-^_ (mmol/l)	26	22-29
Total bilirubin (mg/dl)	1.2	0.0-1.6
Indirect bilirubin (mg/dl)	0.8	0.1-1.2
INR	1	0.9-1.1
PTT (seconds)	28.7	25.7-35.2

**Figure 1:**
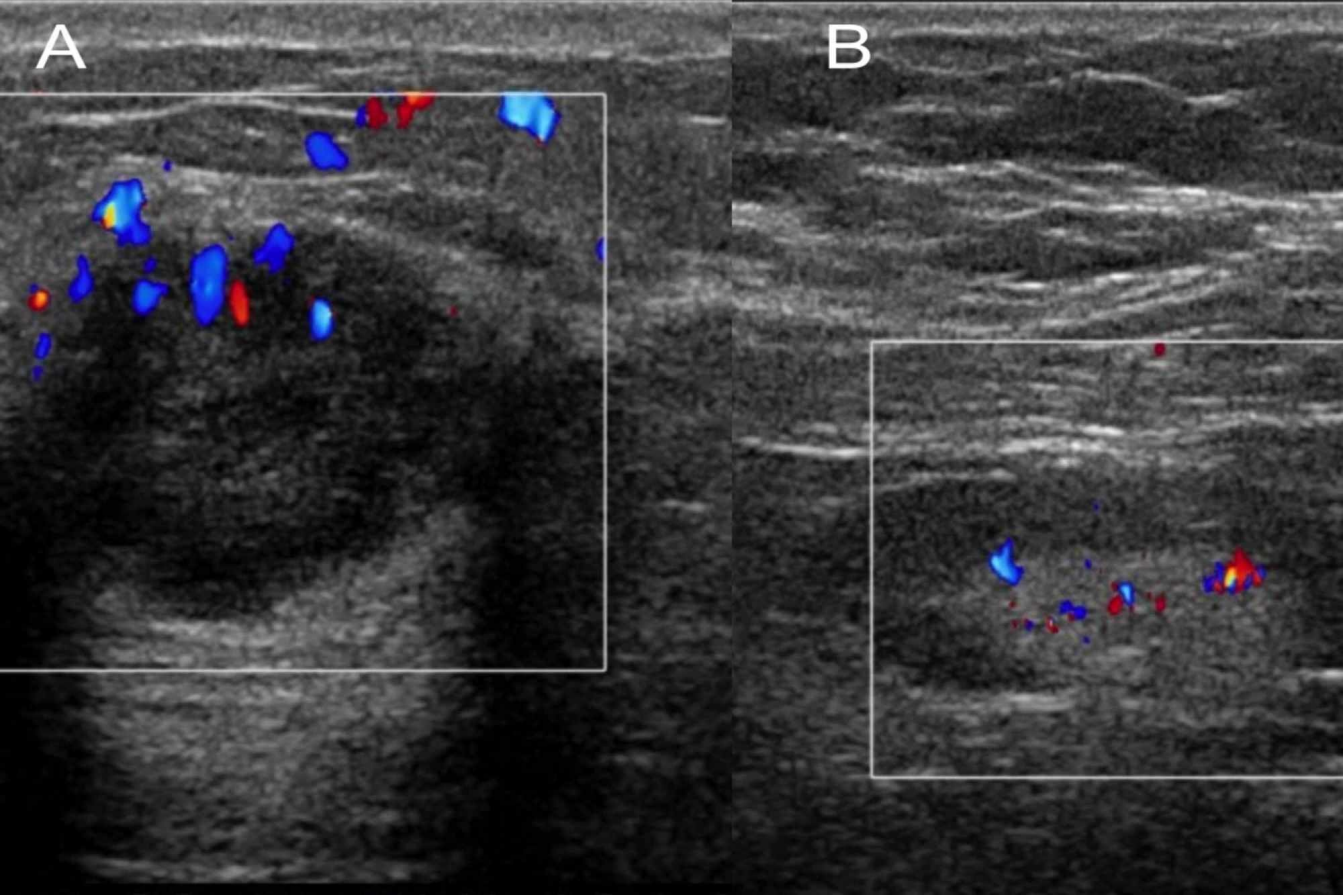
Ultrasound of the right breast and axilla. The ultrasound of the right breast (A) and axilla (B) demonstrates a 1.6 x 1.3 x 1.8 cm microlobulated hypoechoic mass. Several abnormal lymph nodes were noted within the right axillary region with abnormally thickened cortices.

**Figure 2:**
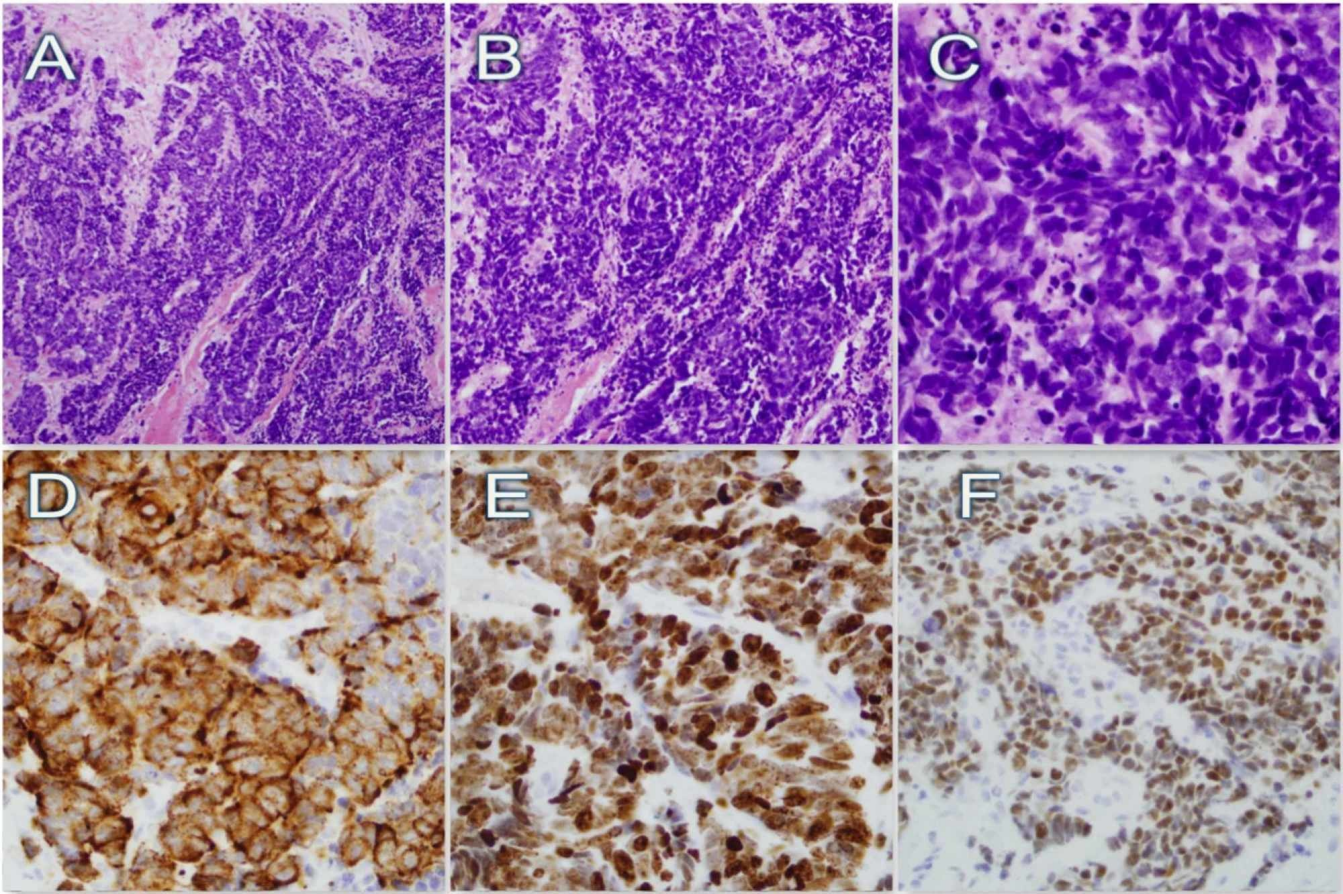
Histological and immunohistochemical findings of the breast mass. A (10x), B (20x), and C (60x) demonstrate a high-grade neoplasm, with highly pleomorphic nuclei, displaying nuclear molding and numerous apoptotic bodies. The cells are small to medium, round to polygonal with inconspicuous nucleoli. D, E, and F demonstrate immunohistochemical stains (chromatin, Ki67, and thyroid transcription factor-1 (TTF-1) respectively). Notably, Ki67 has a proliferative rate of almost 100%.

**Figure 3:**
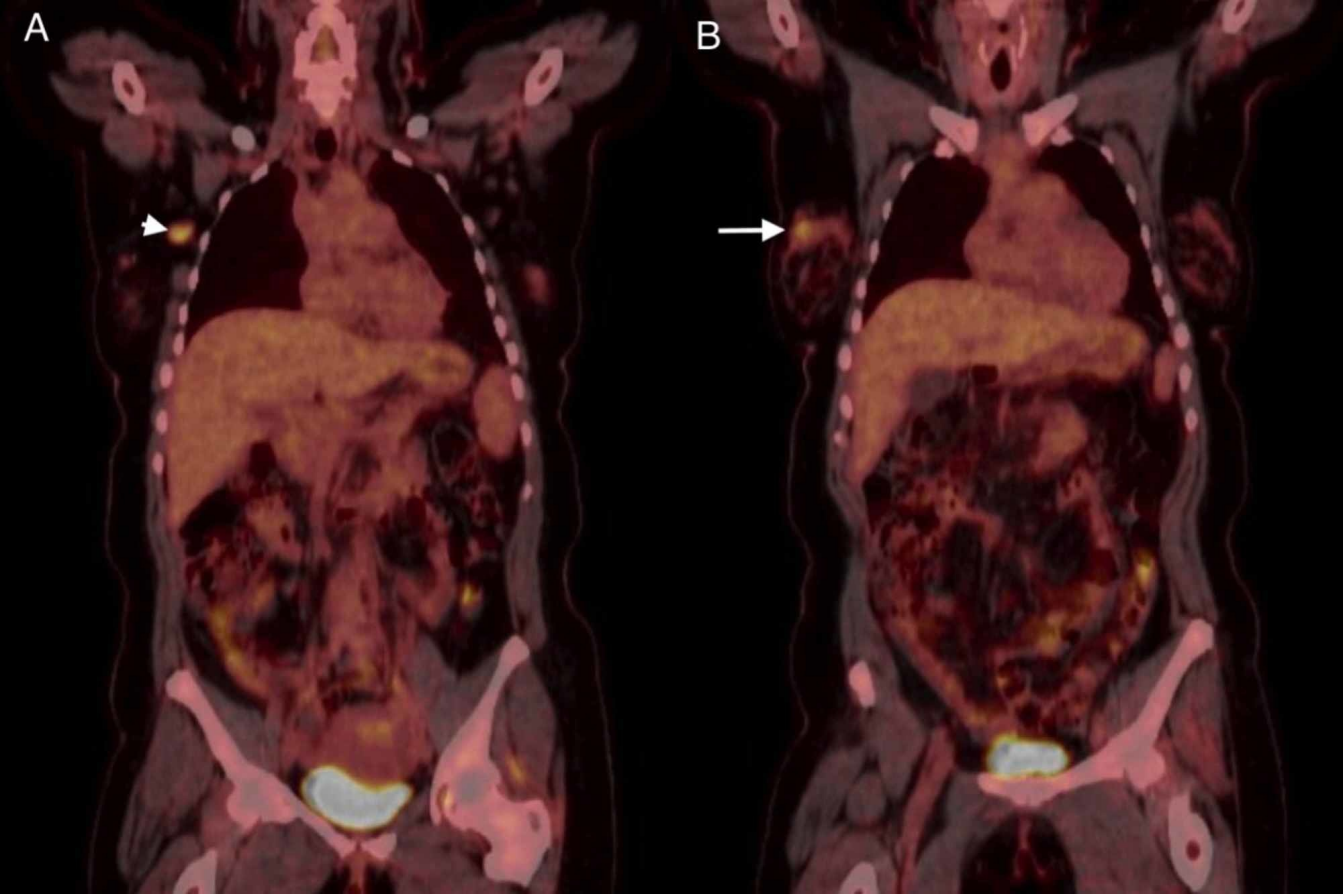
Fluorodeoxyglucose (FDG)-positron emission tomography-computed tomography (PET-CT) of the patient upon initial presentation. (A) The white arrowhead shows a focal region of increased FDG avidity in the lateral aspect of the right breast tissue. (B) The white arrow shows a single enlarged right axillary lymph node visualized posterior to the lateral chest wall measuring up to 2.5 cm in greatest axial dimension with avid FDG uptake.

Ultrasound (US) of the right breast and axilla was suspicious for malignancy (Figure 1), which was followed by an US-guided open-cut biopsy of the right breast. Histology revealed sheets of small oval cells with a high nuclear/cytoplasmic ratio; the nuclei were composed of finely dispersed chromatin, typical for small cell neuroendocrine carcinoma; and the immunohistochemistry showed positivity for chromogranin, synaptophysin, and thyroid transcription factor-1 (TTF-1) (Figure 2). However, estrogen and progesterone receptors were non-reactive. Fine needle aspiration of the right axillary lymph node was performed twice and was negative for malignancy. Positron emission tomography-computed tomography (PET-CT) scan showed focal fluorodeoxyglucose avid right lateral breast mass with two axillary lymph nodes, without any evidence of pulmonary disease (Figure 3). Findings were most consistent with right-sided primary breast cancer and nodal disease without distant metastatic disease. The patient was diagnosed with stage IIIA, T1N2M0, limited, extrapulmonary small cell neuroendocrine carcinoma. Treatment options were discussed in depth, including chemotherapy and radiation therapy. The patient declined all modalities of treatment and preferred to proceed with palliative measures due to religious reasons. 

Eighteen months after diagnosis, the patient presented acutely with altered mental status for 10 days' duration before the presentation. Physical exam revealed stable vital signs though the patient was disoriented. The right breast exam showed enlargement of the tumor with evidence of bleeding. Labs revealed critically severe hypokalemia of 2 mmol/l, sodium of 150 mmol/l, chloride of 99 mmol/l, metabolic alkalosis with bicarbonate level of 34 mmol, normocytic anemia with hemoglobin level of 6.5 g/dl, and a mean corpuscular volume of 83.1 fl (Table 2)

**Table 2:** Lab values on admission to the hospital ANC: absolute neutrophil count; BUN: blood urea nitrogen; Cl^-^: chloride; HCO_^3-^_: bicarbonate; INR: international normalized ratio; K^+^: potassium; MCV: mean corpuscular volume; Na^+^: sodium; PTT: partial thromboplastin time; WBC: white blood cell.

Lab test (unit)	Result	Reference value
Complete blood count		
Hemoglobin (g/dl)	6.5	12-15.5
Hematocrit (%)	19.5	34.9-44.5
MCV (fl)	83	81.6-98.3
WBC (× 10^9^/l)	9	3.5-10.5
ANC (× 10^9^/l)	5.19	1.7-7
Platelets count (x10^9^/l)	230	150-450
Complete metabolic panel and miscellaneous		
Creatinine (mg/dl)	0.7	0.5-1.2
BUN (mg/dl)	10	6.0-20
Na^+^ (mmol/l)	150	136-145
K^+^ (mmol/L)	2	3.5-5.1
Cl^-^ (mmol/l)	99	92-107
HCO_3-_ (mmol/l)	34	22-29
Total bilirubin (mg/dl)	1.3	0.0-1.6
Indirect bilirubin (mg/dl)	1	0.1-1.2
INR	1.1	0.9-1.1
PTT (seconds)	30.2	25.7-35.2

Workup for the etiology of hypokalemia suggested ectopic adrenocorticotropic hormone (ACTH) production, with ACTH level of 247 pg/ml, elevated cortisol level of 51.7 mcg/dl, renin 1.9 ng/dl/hour, suppressed aldosterone with a level of <4 ng/dl, and excess urine potassium excretion. Neither 1 nor 8 mg of dexamethasone suppressed cortisol level. An abdominal CT scan revealed bilateral adrenal hyperplasia, and brain MRI was negative for any pituitary lesions. Spironolactone 100 mg twice a day and ketoconazole 200 mg three times a day were initiated to suppress the mineralocorticoid activity and cortisol production, respectively. Potassium was also aggressively replaced, and the patient was ultimately placed on a scheduled potassium regimen of 60 mEq oral tablets three times a day to maintain her potassium level within the normal range. The patient elected not to undergo any treatment for her malignancy and passed away a few months later. 

## Discussion

EPSCC of the breast is an extremely rare tumor with an incidence of less than 1% of all breast cancers [6]. It has different clinicopathological features and immunohistochemical profile when compared to other types of breast cancer and neuroendocrine tumors (NET). It is reported that the expression of estrogen receptor expression and the steroid sensitivity increase in this type of cysts which alter the pathological and clinical course, especially in cases with excess steroid production [7,8].

Diagnostic Approach

Initial presentation of breast EPSCC is usually a breast lump with or without lymph node involvement that is found on a routine exam or a screening mammogram [4]. It appears as a round, sharply circumscribed, and hyperdense mass on mammography. Reported ultrasound findings include ill-defined margins, enhanced posterior echo, cystic component, and increased vascularity [9]. However, these findings are not specific and may vary between patients [4].

After establishing the presence of a suspicious breast nodule, the diagnostic process usually starts with obtaining an open-cut or surgical biopsy, demonstrating densely packed spindle-shaped cells in a solid and syncytial pattern with a high nuclear to cytoplasmic ratio and mitotic rate with areas of necrosis [10]. Although it shares some histological features to small cell lung cancer (SCLC), it might also appear similar to other types of breast cancer with some degree of neuroendocrine differentiation, making the diagnosis of primary EPSCC challenging [4]. This warrants further immunohistochemical staining for neuroendocrine markers, such as chromogranin, synaptophysin, TTF-1, estrogen receptors, and the absence of HER2 [11,12]. Fine needle aspiration is usually not adequate due to the cytological similarities with other types of breast cancer [9]. Furthermore, to rule out other primary sites and establish the diagnosis of primary breast EPSCC, it is essential to perform a detailed radiological examination with CT and PET scans [4].

Management

There is no universal management strategy for primary breast EPSCC due to the limited reports in the literature. Current treatment regimens are based on sporadic case reports that tackle the aggressiveness of this disease with extensive locoregional control with surgery, radiation, and chemotherapy, preceded by neoadjuvant and/or followed by adjuvant chemotherapy in almost all cases given the recurrent relapse nature of the disease. The majority of the chemotherapy regimens contain platinum agents and etoposide, regimens usually used for SCLC [13]. Therapeutic agents used for invasive breast cancer such as anthracycline and taxanes have also been used [13]. 

Ectopic Production of ACTH

Paraneoplastic syndromes are commonly reported in SCLC. However, due to the sparsity of reports of EPSCC, there are few data on paraneoplastic syndromes in this entity. Generally, due to the similarities between SCLC and EPSCC, it is assumed that the latter carries a similar risk of developing paraneoplastic syndromes, which are typically caused by hormonal production or autoimmune phenomena secondary to the expression of neural antigens from malignant cells [14].

Ectopic ACTH production (EAS) from a non-pituitary tumor is one of the paraneoplastic syndromes associated with SCC. Even though EAS is considered a part of Cushing’s syndrome, EAS, particularly from breast EPSCC, rarely presents with classic cushingoid features of moon facies, central obesity, or hirsutism as initial presentation when compared with other ACTH-producing tumors. Breast EPSCC patients with EAS more commonly present with weight loss, hypokalemia, metabolic alkalosis, and abnormal glucose tolerance, making the diagnosis more challenging and complicating the clinical course of the primary disease. This difference is attributed to the rapid production of ACTH hormone from the aggressive NET of the breast compared to the slower production noticed in pituitary adenomas and other non-pituitary tumors such as SCLC and carcinoid tumors [15-17].

EAS in the setting of breast cancer is extremely rare. To our knowledge, only six cases of breast EPSCC-producing ACTH have been reported in the literature, which made it difficult to predict the prognosis and clinical progression of such a rare condition [7]. Unlike the prognosis of other NETs, which is considered relatively good, EPSCC-producing ACTH is presumed to have a worse prognosis with a more complicated course due to the effect of excess steroid production on the tumor growth, aggressiveness, and complications due to the expression of estrogen receptors. These receptors will be overstimulated in the setting of excess androgen production from the adrenal glands driven by the excess ACTH, and subsequent aromatization of androgens to estrogen, particularly in post-menopausal females [8]

Diagnosis of EAS should be considered in patients with breast EPSCC when they present with signs and symptoms of excess ACTH production such as muscle weakness (82%), hypertension (78%), hirsutism (78%), hypokalemia and metabolic alkalosis (71%), psychiatric disorders (53%), and other less common manifestations [15]. When suspected, 24-hout urinary free cortisol, 1-mg overnight dexamethasone suppression test, and/or late-night salivary cortisol are performed to screen for the condition, followed by measurement of morning serum ACTH levels to establish the diagnosis of ACTH-dependent Cushing's syndrome. Furthermore, 8-mg overnight dexamethasone suppression test, corticotropin-releasing hormone (CRH) stimulation test, and/or inferior petrosal sinus sampling before and after the administration of CRH will help differentiate between ectopic production of ACTH and pituitary adenomas [15].

The optimal therapy of EAS is the surgical removal of the primary tumor. Even if the tumor has metastasized to the liver, resection or cryoablation of the metastases or even liver transplantation may result in a cure [15]. In non-resectable cases, medical adrenal enzyme inhibitors such as metyrapone, ketoconazole, and etomidate are used to achieve clinical suppression of adrenal enzymes and control symptoms. In rare cases, when the disease is indolent with long life expectancy, medical adrenalectomy with mitotane was reported to be effective in patients who are not surgical candidates [15]

## Conclusions

Primary EPSCC of the breast is an extremely rare breast malignancy that can rarely be complicated with the ectopic production of ACTH. Extensive diagnostic evaluation, including histological and immunohistochemical studies in addition to comprehensive metastatic workup, is necessary to differentiate from other primary malignancies such as lung and other breast cancers. Minimal information is reported in the literature regarding the management approach and prognosis, particularly when associated with ectopic hormonal production. Further reports, retrospective, and prospective analyses are warranted to understand the approach and management of this malignancy.

## References

[1] Duguid JB, Kennedy AM (1930). Oat-cell tumours of mediastinal glands. J Pathol Bacteriol.

[2] Frazier SR, Kaplan PA, Loy TS (2007). The pathology of extrapulmonary small cell carcinoma. Semin Oncol.

[3] Feyrter F, Hartmann G (1963). On the carcinoid growth form of the carcinoma mammae, especially the carcinoma solidum (gelatinosum) mammae. Frankf Z Pathol.

[4] Inno A, Bogina G, Turazza M (2016). Neuroendocrine carcinoma of the breast: current evidence and future perspectives. Oncologist.

[5] Brennan SM, Gregory DL, Stillie A, Herschtal A, Mac Manus M, Ball DL (2010). Should extrapulmonary small cell cancer be managed like small cell lung cancer?. Cancer.

[6] Lopez-Bonet E, Alonso-Ruano M, Barraza G, Vazquez-Martin A, Bernado L, Menendez JA (2008). Solid neuroendocrine breast carcinomas: incidence, clinico-pathological features and immunohistochemical profiling. Oncol Rep.

[7] Uchida N, Ishiguro K, Suda T, Horie Y, Nishimura M (2010). ACTH-producing breast cancer: a patient report. Yonago Acta Med.

[8] Wigg SJ, Ehrlich AR, Fuller PJ (1999). Cushing's syndrome secondary to ectopic ACTH secretion from metastatic breast carcinoma. Clin Endocrinol.

[9] Angarita FA, Rodriguez JL, Meek E, Sanchez JO, Tawil M, Torregrosa L (2013). Locally-advanced primary neuroendocrine carcinoma of the breast: case report and review of the literature. World J Surg Oncol.

[10] Chua RS, Torno RB, Vuletin JC (1997). Fine needle aspiration cytology of small cell neuroendocrine carcinoma of the breast. A case report. Acta Cytol.

[11] Adegbola T, Connolly CE, Mortimer G (2005). Small cell neuroendocrine carcinoma of the breast: a report of three cases and review of the literature. J Clin Pathol.

[12] Wang J, Wei B, Albarracin CT, Hu J, Abraham SC, Wu Y (2014). Invasive neuroendocrine carcinoma of the breast: a population-based study from the surveillance, epidemiology and end results (SEER) database. BMC Cancer.

[13] Tremelling A, Samuel S, Murray M (2017). Primary small cell neuroendocrine carcinoma of the breast: a case report and review of the literature. Int J Surg Case Rep.

[14] Gandhi L, Johnson BE (2006). Paraneoplastic syndromes associated with small cell lung cancer. J Natl Compr Canc Netw.

[15] Ilias I, Torpy DJ, Pacak K, Mullen N, Wesley RA, Nieman LK (2005). Cushing’s syndrome due to ectopic corticotropin secretion: twenty years’ experience at the National Institutes of Health. J Clin Endocrinol Metab.

[16] Poddar NK, Saha R, Hedau S, Ray A (2005). Adrenocorticotropic hormone production in breast cancer. Indian J Exp Biol.

[17] Isidori AM, Kaltsas GA, Pozza C (2006). The ectopic adrenocorticotropin syndrome: clinical features, diagnosis, management, and long-term follow-up. J Clin Endocrinol Metab.

